# Highlight: Lost Traits and the Persistence of Complexity

**DOI:** 10.1093/gbe/evae101

**Published:** 2024-05-21

**Authors:** Casey McGrath

Traits are often lost during evolution, either because they are no longer beneficial or because they are too costly to maintain. When this happens, it is generally believed that the genes underlying the trait will eventually degrade as well, making it difficult if not impossible for the trait to re-emerge. Yet, there are numerous examples in nature of once-lost traits reappearing in descendent lineages. According to Giobbe Forni, a Research Fellow at the University of Bologna, “Mapping the presence and absence of traits onto a species tree suggests that some traits may have been lost in the lineages leading to extant species and then subsequently reinstated. Wings in stick insects are considered one of the more iconic instances of this evolutionary process.” This implies that the genes underlying these traits may be preserved, in some cases for millions of years. Unfortunately, research on the molecular basis of such re-emergence is sparse, leaving the underlying mechanisms responsible for such preservation largely open to speculation until now. In a new study published in *Genome Biology and Evolution*, Forni and his colleagues shed light on another complex trait that has been lost in some stick insects—the production of males (Forni et al. 2024). Loss of the ability to produce males results in populations of only females, which reproduce by parthenogenesis, a form of asexual reproduction. The study reveals that genes that are highly connected in regulatory networks and involved in multiple biological processes may be maintained long after a trait is lost, providing a potential avenue for trait re-emergence over long evolutionary time scales.

**Fig. 1.**
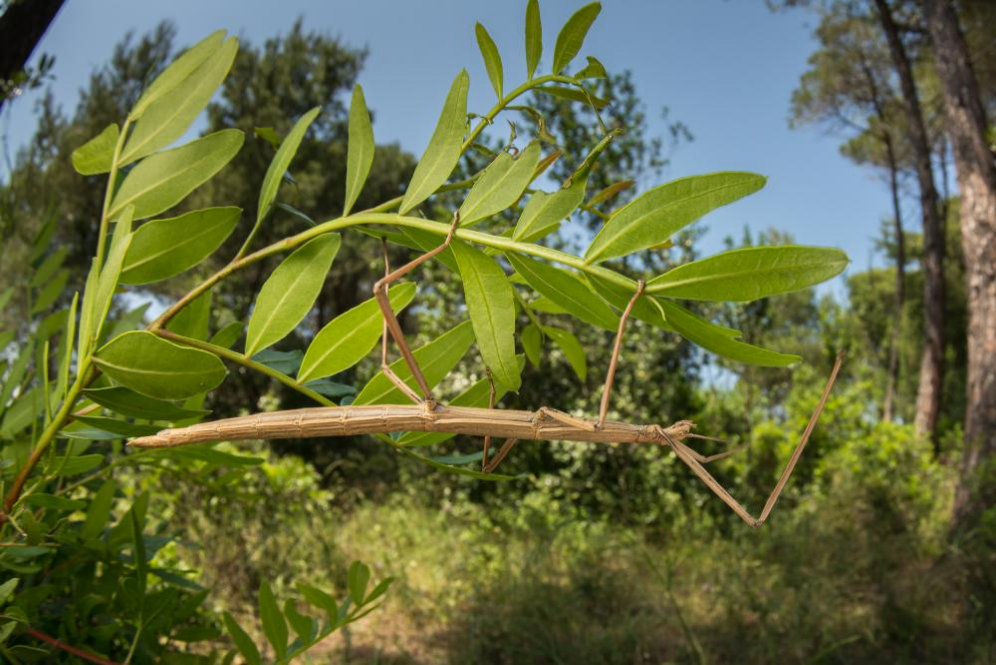
Photograph of a stick insect. Credit: Filippo Castellucci.

In the new study, Forni and his co-authors Barbara Mantovani, Alexander S. Mikheyev, and Andrea Luchetti performed a comparative analysis of three species of stick insects in the genus *Bacillus* (Fig. 1). While *Bacillus grandii marettimi* populations are composed of males and females that reproduce sexually, *Bacillus atticus* populations have lost the ability to produce males, comprising only females that reproduce by parthenogenesis. A third species, *Bacillus rossius*, includes both sexual populations and parthenogenetic populations that have lost the ability to produce males. By studying the fates of genes involved in male reproduction in these three species, the authors sought to investigate the extent to which genes are preserved after trait loss and the potential mechanisms driving this preservation.

The researchers first identified gene networks whose expression was correlated with either male or female reproduction in the sexual species *B. marettimi* and then evaluated the same genes in *B. atticus* and *B. rossius*. Surprisingly, male-related genes exhibited no signs of weakened selection or accelerated evolution compared with female-related genes in the parthenogenetic species. Furthermore, male-related patterns of gene expression were partially preserved across both parthenogenetic species.

Delving deeper, the researchers found that genes in female-related networks were primarily expressed in female reproductive tissues, while those in male-related networks were expressed in male *and* female reproductive tissues, including both sexual and parthenogenetic females. This suggests that male-related genes may also play roles in female reproduction. The involvement of a gene in multiple biological processes is known as pleiotropy, and this phenomenon may explain the preservation of male-related genes in these parthenogenetic stick insects, as previously hypothesized.

Moreover, the authors found that genes that were highly connected to many other genes in the network were more likely to be expressed in the reproductive tissues of parthenogens, suggesting that a gene's network connectivity may also influence its gene preservation after trait loss. Taken together, these findings indicate “that the molecular ground plan of the once-lost male reproductive process may persist due to pleiotropic effects on other traits,” explains Forni. “Different genes may undertake different trajectories of preservation and decay depending on the level of pleiotropy within the gene regulatory network.”

This study not only sheds light on genetic architecture persistence after trait loss but also offers a potential glimpse into the emergence of rare males and cryptic sex (i.e. episodic generation of males and sexual reproduction), which have been observed in an increasing number of lineages that were thought to have lost the ability to produce males long ago. This opens up new potential avenues for research, with implications that may reach far beyond stick insects. “Looking at how widespread genetic preservation after trait loss is on a larger scale remains fundamental. Although the *Bacillus* species complex offers a nice framework to address these issues, it would be useful to analyze a larger species complex where multiple transitions between reproductive strategies has occurred,” notes Forni. “While it is often necessary to rely on model species to discover and dissect biological processes, it is even more important to test our hypotheses in a wider context. This will be possible only if we dedicate more effort to observing and analyzing the amazing diversity of organisms and their intricate adaptations.”

***Want to learn more?*** Check out these other articles from *Genome Biology and Evolution* on the evolutionary consequences of sex loss in insects:

1. Contrasting evolutionary patterns between sexual and asexual lineages in a genomic region linked to reproductive mode variation in the pea aphid (Rimbault et al. 2023)
2. Divergent gene expression following duplication of meiotic genes in the stick insect *Clitarchus hookeri* (Wu et al. 2021)
3. Genome of the parasitoid wasp *Diachasma alloeum*, an emerging model for ecological speciation and transitions to asexual reproduction (Tvedte et al. 2019)
4. Decay of sexual trait genes in an asexual parasitoid wasp (Kraaijeveld et al. 2016)
